# “Those pot heads” – perceived external stigma and self-stigma among cannabis users in Germany: prevalence and associations with socio-demographics, cannabis use patterns and psychological distress

**DOI:** 10.1186/s42238-025-00328-1

**Published:** 2025-09-17

**Authors:** Moritz Rosenkranz, Anna Schranz, Uwe Verthein, Georg Schomerus, Sven Speerforck, Jakob Manthey

**Affiliations:** 1 Center for Interdisciplinary Addiction Research (ZIS), Department of Psychiatry and Psychotherapy, University Medical Center Hamburg-Eppendorf (UKE), Martinistraße 52, 20246 Hamburg, Germany; 2 Department of Psychiatry, Medical Faculty, University of Leipzig, Semmelweisstr. 10, 04103 Leipzig, Germany

**Keywords:** External stigma, Self-stigma, Cannabis use, Cannabis legalization

## Abstract

**Background:**

Cannabis users have been stigmatized in the course of the long-standing prohibition. A recent law change in Germany made cannabis possession and cultivation legal for recreational use, potentially impacting stigmatization. This article aims to quantify the experience of perceived and self-stigma related to cannabis use before the law change and to explore associations with sociodemographic-, cannabis use pattern- and psychological distress-related items.

**Methods:**

Participants were recruited in 2023 from ISO-certified online access panels. A subsample of *n* = 684 regular (at least monthly) cannabis users was selected through quota-sampling based on age, gender, education, and federal state to reflect the demographic composition of the German population aged 18–64. A standardized online-questionnaire covering sociodemographics, health-related variables, cannabis use (frequency, purpose) as well as experiences of external and self-stigmatization was employed. Descriptive data analyses were performed utilizing the stigma-related items as outcome variables.

**Results:**

External Stigmatization was perceived by 30.6%, while 22.1% reported self-stigma concerning their cannabis use. Higher education, high psychological distress, medical use, and cannabis use disorder (CUD) were significantly associated with both external stigmatization and self-stigma. Respondents speaking publicly about their cannabis use show higher shares of external and self-stigma. Having been in trouble with the police regarding cannabis was positively associated with both types of stigmatization.

**Conclusion:**

German residents who use cannabis at least monthly perceived external stigmatization and self-stigma related to cannabis consumption at a relevant level. As medical users and those with CUD are particularly affected, healthcare providers should be sensitized to the issue of stigmatization.

## Background

In 2014, Uruguay became the first country in the world to legalize the possession and home cultivation of recreational cannabis as well as the establishment of cannabis social clubs which are allowed to provide self-cultivated cannabis to club members. In the following years, several US states also legalized cannabis and in 2018, a respective change in the law took place in Canada, making it the first country of the Organization for Economic Co-operation and Development (OECD) to legalize recreational cannabis retail at state level. More recently, Germany has legalized cannabis possession as well as personal and collective cultivation, yet commercial retail remains prohibited (Manthey et al. 2024).

Although the law changes have made cannabis legal for recreational use, the question arises as to whether and to what extent cannabis use is socially legitimized as a result. Concomitant with the prohibition of most common psychoactive substances around 100 years ago, people who use cannabis (PWUC) were increasingly stigmatized as lazy, criminal, amoral, and a menace to society (Gasnier et al. 1936; Stringer and Maggard 2016). This is also reflected in pejorative terms such as “pot head” or “stoner” (Erving 2012), which were used in an attempt to ridicule PWUC and marginalize them in society. This negative image of cannabis use was subsequently also adopted by PWUC themselves, on the one hand as a resistance in the sense of empowerment and reappropriation of the term as a self-description (Galinsky  2003). But more often the terms are likely to have led to PWUC themselves perceiving their consumption as something to be ashamed of (which did not lead them to stop using, but to conceal use) (Matthews et al. 2017). Two important dimensions of the "stigmatization" concept are addressed here: External stigmatization and self-stigma. According to Goffman's social stigma theory (Goffman 1963), stigma refers to a characteristic, behavior, or reputation that causes social disapproval in some way. External or public stigma implies that other people label the person in question with undesirable and rejected stereotypes. Closely related to this is the concept of perceived external stigmatization, which addresses how each individual believes that society views him or her personally because he or she is a member of the stigmatized group (LeBel 2008). If an individual recognizes negative public attributions, concurs to them and finally internalizes them, this is referred to as self-stigma (Bathje and Marston 2014).

A lot of research on cannabis and stigma concerns the stigmatization of medical cannabis use(rs) and associated barriers to utilize health care (see e.g. (Fehr et al. 2024; Bottorff et al. 2013)). Some studies examined stigmatization as a risk factor for cannabis use. For example, in a Canadian survey of high school students several stigma markers were assessed regarding their association with cannabis use. Not living with their mother (OR = 1.94), low maternal education (OR = 2.14), and being a member of the indigenous community (OR = 3.38) enhanced the probability of using cannabis, while low socioeconomic status showed no relevant effect (Buttazzoni et al. 2020). Only few studies have investigated perceived external or self-stigma as a consequence of recreational cannabis use. A national survey from Uruguay revealed that obtaining cannabis from legal sources reduced external stigmatization and a higher frequency of use enhanced stigmatization. Employing gender, age and race did not lead to significant results (Queirolo et al. 2023). A recent study from Canada yielded similar results (Coles et al. 2024). Skliamis and colleagues (Skliamis et al. 2020) investigated on the external stigma in seven European countries and reported that perceived devaluation (e. g. PWUC are dangerous or unreliable) was the dimension of stigma that was most commonly reported. Male gender, daily cannabis use, and living a in a country with a rather strict cannabis policy ( i. e. recreational use and possession for personal use is illegal, rigorous sentencing practice on cannabis supply) was associated with a higher probability of perceived devaluation, while sociodemographic factors like age, living situation or employment status did not contribute significantly. Hathaway et al. used in-depth interviews to collect information on self-stigma and reported about internalization of mainstream perspectives on cannabis use as risky or deviant resulting in feelings of guilt (Hathaway et al. 2011). Other studies report that self-stigma correlated positively with the use severity (Elkalla et al. 2023; Melchior et al. 2019), with female gender, younger age and higher scores on depression (Melchior et al. 2019). A systematic review on substance use related perceived stigma identified lower levels of education, female gender, severity/frequency of drug or alcohol use, and lower psychological wellbeing being associated with perceived stigma (Kulesza 2013).

The discussion about the pros and cons of legalizing cannabis for recreational use was very controversial in Germany before the law was changed, with public opinion being rather critical (Klosterkötter and Kuhn 2024; Manthey 2023). In general, there seems to be skepticism in Germany towards a re-evaluation of (currently) illegal drugs, including, for example, their use in a psychotherapeutic setting (Herwig et al. 2024; Perez Rosal et al. 2024).

However, imminent changes in cannabis legislation in Germany (as in other countries) are likely to have an impact on attitudes toward cannabis use. The existing research on the question of whether legislation has an influence on the attitudes and social norms of the population is inconsistent. Although basic attitudes and social norms often change only very slowly (Jost et al. 2009), governmental legislation in democracies is generally perceived as the will of the population, Ofusu and colleagues claim in a study about changes after the legalization of same-sex marriage. They further conclude, that “government legislation can inform individuals’ attitudes, even when these attitudes may be deeply entrenched and socially and politically volatile” (Ofosu et al. 2019). A change in the law (e.g. with regard to Germany: changing the classification of cannabis as an illegal drug to a legal drug) could therefore also change the attitude of the general population as a whole. It is plausible to assume that there could also be underlying circularity here. Changes in attitudes in an increasing proportion of society can lead to laws being changed. The change in laws can in turn further promote the change in attitudes.

Taking these considerations and the results from research on Stigma presented above into account, the aim of the present article is to use baseline data collected before the change in the law in Germany to 1) quantify the experience of perceived and self-stigma in a sample of cannabis users in Germany and 2) to show exploratory associations with sociodemographic-, cannabis use pattern- and psychological distress-related items.

## Methods

### Participants and data collection

The data were collected in November and December 2023 from a sample of *n* = 684 participants who reported using cannabis at least monthly. The criterion of at least monthly cannabis use was adopted here, because the data originate from the larger research project “CannaStreet”. Details about the power calculation for CannaStreet are provided in the corresponding protocol (Schranz et al. 2024b). In the CannaStreet project, a total of *n* = 8556 participants (“basic sample”), aged 18 to 64 years and residing in Germany, were screened for cannabis use using an online retrospective survey conducted via computer-assisted web interviewing (CAWI). The basic sample was selected through quota-sampling based on age, gender, education, and federal state in order to reflect the demographic composition of the German population aged 18–64. Participants who indicated that they use cannabis at least monthly were subsequently invited to complete the questionnaire (“target sample”). The participants were sourced from actively recruited ISO-certified online access panels that engage individuals through both online and offline channels. Participants were compensated financially for their participation.

### Materials

The questionnaire included socio-demographic and health-related variables, questions on cannabis use, a screening for cannabis use disorder (CAST; (Legleye et al. 2007)) and questions on experiences of stigmatization. We also asked whether the respondents usually speak publicly about their use and further, if there had ever been any problems with the police due to cannabis. The complete questionnaire is accessible on OSF (Schranz 2024a).

#### Socio-demographic information

We collected data on gender (female/male/diverse), age groups (18–24/25–34/35–44/45–64 years), level of education (low/mid/high; aggregation of school degrees and occupational levels from ISCED, (OECD 2015)), and migration background (at least one parent or respondent was born abroad) to gain information on the socio-demographic background of participants.

#### Stigma

Perceived stigma was measured using the self-developed item “If other people find out about my cannabis use, they think badly of me “. Self-stigma (in the sense of anticipated shame) due to cannabis use was measured using the self-developed item “I feel ashamed when other people find out about my cannabis use”. Both items were answered on a 5-Point Likert Scale, ranging from strongly disagree to strongly agree. For analysis, the scales of the Stigma items were categorized into 3 groups reflecting disagreement (1 to 2 points), indecisiveness (3 points) and agreement (4 to 5 points).

#### Psychological distress

The level of psychological distress (low (0–7)/moderate (8–12)/high (13–24)) was assessed via Kessler Psychological Distress Scale (K6) (0–24) (Kessler et al. 2002). The K6 scale was designed as a nonspecific instrument for mental health screening in population-based surveys, consisting of 6 items that assess the extent of anxiety and depressive symptoms experienced by individuals over the previous four weeks (Yiengprugsawan et al. 2014).

#### Cannabis use

A screening for cannabis use disorder (CUD) was conducted employing the CAST (Legleye et al. 2007; Legleye 2018). The CAST is a widely used screener with questions on consuming before midday or when being alone, and questions on problems related to cannabis use (memory, unsuccessful quitting, problems at work or accidents) which are to be indicated on a 5-point scale (0 = ”never” to 4 = ”very often”). To determine severe CUD the suggested cut-off ≥ 7 was used.

We further collected data on purpose of cannabis use (medical only/medical and recreational/recreational only), prescription of medical cannabis (only interviewees reported using cannabis (at least partly) medically were asked for a prescription), frequency of speaking publicly about consumption (never/rarely/sometimes vs. (very) often), and having experienced legal problems due to cannabis.

The selection of variables was based on theoretical considerations about stigma and empirical results from previous studies investigating the stigmatization of people with mental illness and/or drug use.

### Data analyses

Due to the explorative character of this contribution, descriptive data analyses were carried out using the two Stigma-related outcome variables, which were correlated with the 10 variables described above.

Bivariate analyses of 10 categorical independent variables were tested for statistical significance using Pearson´s Chi^2^. To control the false discovery rate (FDR) across multiple tests, the Benjamini–Hochberg procedure was applied. Sorted *p*-values were compared to critical values calculated as (rank of *p*-value/total number of tests) x α, with α = 0.05 and total number of tests = 20, and results were deemed significant if the *p*-value was less than or equal to its corresponding threshold. All statistical analyses were conducted using SPSS 27 software (IBM Corp 2020).

## Results

About two-thirds (67.7%) of the sample identified themselves as male, and 31.7% as female gender. The mean age in the sample was 35.9 years (SD: 10.7y). Furthermore, a rather high level of education was found in the total sample, with only 5.4% of respondents belonging to the group with a low educational level, and more than half (56.0%) have a high level of education. 16.8% reported a migration background, meaning that the respondents themselves or at least one parent were born abroad. Lastly, about one quarter of the sample is highly burdened by psychological distress. Table 1 further shows that around one-third (30.6%) of the respondents stated that they perceived stigmatization and about one fifth (22.1%) reported experiencing self-stigma concerning their cannabis use.

**Table 1:** Sample characteristics

		n	%
Perceived stigmatization	yes	209	30.6
	neither/nor	219	32.0
	no	256	37.4
Self-stigma	yes	150	22.1
	neither/nor	173	25.5
	no	356	52.4
Gender	female	217	31.7
	male	463	67.7
	diverse	4	0.6
Age group (years)	18–24	84	12.3
	25–34	263	38.5
	35–44	203	29.7
	45–64	134	19.6
Level of education	low	37	5.4
	mid	263	38.6
	high	382	56.0
Migration background	yes	115	16.8
	no	568	83.2
Psychological distress (categorized)	low	293	42.8
	moderate	216	31.6
	high	175	25.6
Purpose of cannabis use (past 12 months)	medical only	157	23.0
	medical and recreational	208	30.4
	recreational only	319	46.6
Prescription of medical Cannabis	yes	218	59.7
	no	147	40.3
Severe cannabis use disorder (acc. to CAST; cut-off ≥ 7)	yes	411	61.0
	no	263	39.0
Public speaking about cannabis use (never/rarely/sometimes vs. (very) often; without "don't know")	yes	535	80.2
	no	132	19.8
Ever been in trouble with police in connection with cannabis use (without "don´t know")	yes	154	23.3
	no	506	76.7

Regarding the purpose of cannabis use, almost half of the sample (46.6%) indicated recreational use only while 23.0% use cannabis for medical purposes exclusively. A physician's prescription for medical cannabis was reported by 59.7% of interviewees. More than half of respondents (61.0%) screened positive on the CAST, indicating a high proportion of severe CUD in the sample. A majority (80.2%) reported to speak publicly about their cannabis use and 23.3% ever had trouble with the police in the context of cannabis.

Table 2 shows the correlation of perceived and self-stigma experiences with socio-demographics and psychological distress. We found no significant differences in stigma experiences regarding gender but perceived (39.9%) as well as self-stigma (28.6%) appeared to be more prevalent in the age group of 25–34-year-olds. Further, greater shares of people with a high level of education report stigmatization of both types than people from groups with middle or low level of education. Respondents who indicated having a migration background were also more frequently affected by both external and self-stigmatization. People reporting high psychological distress indicated significantly more often perceived (46.9%) as well as self-stigma (39.3%) compared to respondents with moderate or low psychological distress.

**Table 2:** Sociodemographics and psychological distress (row percent) by type of stigmatization

		Perceived stigmatization				Self-stigma				
		yes	n/n	no	Sig	yes	n/n	no	Sig	n
Gender, %	female	28.1	30.4	41.5	χ^2^ = 2.033; *p* = 0.362	18.1	25.6	56.3	χ^2^ = 3.098; *p* = 0.212	217
	male	31.5	32.6	35.9		23.9	25.4	50.7		463
Age group (years), %	18–24	25.0	29.8	45.2	χ^2^ = 22.523; *p* < 0.001*	20.7	24.4	54.9	χ^2^ = 22.347; *p* = 0.001*	84
	25–34	39.9	30.8	29.3		28.6	27.5	43.9		263
	35–44	26.6	34.5	38.9		19.7	27.6	52.7		203
	45–64	21.6	32.1	46.3		13.6	18.9	67.4		134
Level of education, %	low	10.8	48.6	40.5	χ^2^ = 17.924; *p* = 0.001*	10.8	16.2	73.0	χ^2^ = 34.365; *p* < 0.000*	37
	mid	25.9	30.8	43.3		15.6	21.0	63.4		263
	high	35.9	30.9	33.2		27.8	29.6	42.6		382
Migration background, %	yes	31.9	29.6	38.6	χ^2^ = 9.621; *p* = 0.008*	23.4	23.9	52.7	χ^2^ = 5.944; *p* = 0.051	568
	no	24.3	44.3	31.3		15.9	33.6	50.4		115
Psychological distress, %	low	21.8	26.6	51.5	χ^2^ = 59.771; *p* < 0.000*	12.4	15.8	71.8	χ^2^ = 95.833; *p* < 0.000*	293
	moderate	29.2	38.0	32.9		21.4	33.5	45.1		216
	high	46.9	33.7	19.4		39.3	31.8	28.9		175

Sig: Significance tests. n/n: neither/nor. As gender "diverse" only refers to *n* = 4, these cases were set to missing value in this analysis. *P*-values are considered significant after controlling the false discovery rate (FDR) using the Benjamini–Hochberg procedure (α = 0.05). Asterisks (*) indicate FDR-significant results

Table 3 shows how stigma experiences are linked to medical use of cannabis. Almost half of respondents (47.1%), who use cannabis only for medical purposes, experienced perceived stigmatization by others and self-stigma (41.9%). The perceived stigmatization and self-stigma are lowest among exclusively recreational users. Respondents with a physician´s prescription of medical cannabis more often report experiences of both types of stigma as compared to those without a prescription.

**Table 3:** Purpose of Cannabis use and medical prescription (row percent) by type of stigmatization

		Perceived stigmatization				Self-stigma				
		yes	n/n	no	Sig	yes	n/n	no	Sig	n
Purpose of cannabis use (past 12 months), %	medical use only	47.1	19.1	33.8	χ^2^ = 35.531; *p* < 0.000*	41.9	21.3	36.8	χ^2^ = 49.280; *p* < 0.000*	157
	medical and recreational use	28.4	39.4	32.2		17.9	29.5	52.7		208
	recreational use only	23.8	33.5	42.6		15.1	24.9	59.9		319
Prescription of medical Cannabis, %	yes	42.2	26.6	31.2	χ^2^ = 8.337; *p* = 0.015*	37.0	27.3	35.6	χ^2^ = 27.467; *p* < 0.000*	218
	no	27.9	36.7	35.4		15.1	24.0	61.0		147

Sig: Significance tests. n/n: neither/nor. Medical cannabis = flowers or proprietary medicinal products such as Sativex or Dronabinol. *P*-values are considered significant after controlling the false discovery rate (FDR) using the Benjamini–Hochberg procedure (α = 0.05). Asterisks (*) indicate FDR-significant results

Comparisons between stigmatization by others and self-stigma regarding further cannabis-related items are presented in Table 4. Interviewees, who showed a severe CUD according to the CAST screener, perceived significantly more often external stigmatization (38.4%) than people without CUD (18.3%). The same applies to self-stigma (28.7% vs. 10.7%). Speaking publicly about cannabis use is associated more often with having perceived stigmatization by others (54.5%), but also with self-stigma (42.4%). People having been in trouble with the police due to cannabis use, more frequently reported perceived stigmatization (43.5%) and self-stigma (29.2%) compared to people who never had an encounter with the police for cannabis.

**Table 4:** Cannabis use disorder, public speaking about use, and legal problems due to Cannabis (row percent) by type of stigmatization

		Perceived stigmatization				Self-stigma				
		yes	n/n	no	Sig	yes	n/n	no	Sig	n
Severe CUD (acc. to CAST; cut-off ≥ 7), %	yes	38.4	36.7	24.8	χ^2^ = 75.930; *p* < 0.000*	28.7	31.2	40.0	χ^2^ = 70.219; *p* < 0.000	411
	no	18.3	24.0	57.8		10.7	16.4	72.9		263
Public speaking about Cannabis, %	yes	54.5	18.2	27.3	χ^2^ = 45.265; *p* < 0.000*	42.4	25.0	32.6	χ^2^ = 44.111; *p* < 0.000*	132
	no	24.7	35.5	39.8		16.8	25.8	57.4		535
Trouble with police in connection with cannabis use	yes	43.5	31.2	25.3	χ^2^ = 18.187; *p* < 0.000*	29.2	26.6	44.2	χ^2^ = 7.832; *p* = 0.020*	154
	no	27.1	31.8	41.1		19.9	24.5	55.7		506

Sig: Significance tests. n/n: neither/nor. *CUD* cannabis use disorder. *P*-values are considered significant after controlling the false discovery rate (FDR) using the Benjamini–Hochberg procedure (α = 0.05). Asterisks (*) indicate FDR-significant results

## Discussion

Results show that both, perceived Stigmatization and self-stigma concerning cannabis use are relevant issues in this sample of German residents using cannabis at least monthly. Other studies reported higher numbers on slightly different stigma scenarios and regarding drugs in general. In a sample of adolescents, 54% would feel ashamed if someone knew about a drug addict in the family (Adlaf et al. 2009). Among drug users (cocaine/heroin) from New York City, the prevalence of perceived stigma (devaluation) was 85% (Ahern et al. 2007). A similar share (86%) of at-risk drinkers from 6 southern US-states indicated to have perceived stigma related to their alcohol use. Although the existing evidence shows that stigmatization is generally lower for legal substances such as alcohol and, in some US-states, cannabis (Kulesza 2023; Krendl and Perry 2023; Palamar et al. 2012), the possession of recreational cannabis was not yet legal in Germany at the time of data collection. However, the announced legalization may already have had a corresponding effect here.

Higher educated PWUC in our sample are significantly more often affected by public stigmatization as well as self-stigma. It is conceivable that the stereotype of a lazy “pot head” which didn´t achieve any occupational goals is driving both types of stigmatization here, possibly because the cannabis stereotype stands out more strongly against people with higher education and thus induces stigmatization.

The higher prevalence of perceived and self-stigmatization among people with a migration background could be related to the fact that immigrants are exposed to social and media stigmatization in many ways and are often suspected of engaging in illegal activities (Albrecht 2016; Kakavand and Trilling 2022).

The proportion of cannabis users with high psychological distress is more than two times higher than in the group with low distress regarding perceived stigmatization, and more than three times higher when it comes to self-stigma (39.1% vs. 12.4%). Due to the explorative, descriptive character of this contribution, it remains unclear if people burdened with psychological distress perceive stigmatization more quickly respectively tend to stigmatize themselves more strongly or whether the psychological distress is a consequence of the stigmatization. A reciprocal effect is also plausible.

Respondents who use cannabis for medical reasons as well as those who have a physician´s prescription reported both types of stigmatization to a much greater extent than recreational users or those indicating both use purposes. This is an interesting result, because these people are using an authorized medicine. The public stigmatization of treatment with medical cannabis (see e.g. (Satterlund et al. 2015) may still have to do with the widespread public perception of cannabis as an illegal drug and not a medicine, but the reason for self-stigma in this regard remains unclear. One possible explanation would be the adoption of the negative public perception into the self-conception and people feel ashamed to use a “drug” to seek relieve from symptoms and not a “medicine”.

Higher shares of both perceived stigmatization and self-stigma in the group with severe CUD according to CAST may be based on a similar mechanism. The negative attitudes towards addiction that prevail in considerable parts of society (Krendl and Perry 2023; Matthews et al. 2019; Yang et al. 2017) are not only reflected in the perception of public stigmatization, but – according to our results – also generate feelings of shame among those affected by the problem—similar to cannabis as a medicine.

Given the findings on stigmatization of drug users presented above, we expected that public talk about cannabis would promote stigmatization. As previous studies have shown, being recognized as a drug user can initially result in external stigmatization, and subsequently to internalization of the norm in the sense of self-stigmatization (Matthews et al. 2017). We see this phenomenon in our sample, too, where talking about cannabis in public is associated with significantly higher levels of self-stigma compared to respondents who conceal their use and are less likely to face possible negative opinions about cannabis from others.

Users who have already been in trouble with the police for cannabis reported to a greater extent about perceived stigmatization, but not so much about self-stigma. It is possible that the experience of stigmatization relates to the encounter with the police or a negative interaction with the person by the police. This result can also be interpreted as a further hint that a prohibitive approach to cannabis use can have negative consequences for treatment seeking and utilizing behavior. This is because the experience that users are stigmatized when external institutions find out about their use could also lead to concerns about making use of counselling and treatment services. Apart from this, trust in the police in general could be diminished, as the overall low levels of self-stigmatization indicate that few individuals tend to regard their cannabis use as something to be ashamed of or even a criminal offence. However, the lower levels of self-stigma among people having had trouble with the police might mirror an externalization (rather than internalization) of stigma, when the police is perceived as “the others” whose attitudes have little relevance to how the person sees itself.

## Limitations

Some limitations must be taken into account when interpreting the results. Firstly, to assess both, perceived external stigmatization and self-stigma due to survey constraints only one item was employed for each of them. Both types of stigmatization are multilayered constructs, so this approach may not reflect the actual complexity. Given the lack of empirical data regarding stigmatization of cannabis use, our approach still provides valuable insights on this topic. However, it would be beneficial if psychometrically validated measurement instruments were developed in the future to further improve the quality of evidence on stigmatization related to cannabis use.

Further, we found considerable proportions of respondents having chosen the “neither/nor” option of the stigma items. It remains unclear whether these people are indifferent to the issue of stigmatization, in which case they would be more likely to be classified as non-stigmatized. On the other hand, it would also be conceivable, that these people are willing to tolerate more negative reactions to their consumption before claiming to have been stigmatized. In this case, they would belong to the stigmatized group. Because of this ambiguity, the middle was retained as a separate category.

## Conclusion

Perceived external stigmatization as well as self-stigma is a common experience among cannabis users, especially among medical users and those affected by CUD. This might pose a barrier to utilize specific counselling or treatment, for example due to shame (Hammarlund et al. 2018). Anti-stigma campaigns targeting especially treatment providers and other health professionals could be useful, because stigmatization is also a relevant phenomenon in this occupational group, with potential negative impact on adequate treatment (Boekel et al. 2013).

Our findings might be used to generate specific hypotheses or research questions for further studies on Stigmatization of PWUC. With a repeated cross-sectional study design, we will monitor the development of cannabis stigma in the German population and assess the possible impact of the 2024 legalization. Nevertheless, there is a need for further comparative studies to contrast the present findings.

## Data Availability

The datasets generated and/or analysed during the current study are not publicly available due to contractual restrictions. The complete questionnaire of the research project “CannaStreet” is accessible on OSF.

## Abbreviations

CAST: Cannabis Abuse Screening Test
CAWI: Computer-assisted Web Interviewing
CUD: Cannabis use disorder
ISCED: International Standard Classification of Education
K6: Kessler Screening Scale for Psychological Distress
OECD: Organization for Economic Co-operation and Development
OR: Odds Ratio
OSF: Open Science Framework
PWUC: People who use cannabis
SPSS: Statistical Package for the Social Sciences
